# The association between intracompartmental pressure and skeletal muscle recovery after tibial diaphyseal fractures: an ambispective cohort study

**DOI:** 10.1186/s10195-021-00579-7

**Published:** 2021-05-06

**Authors:** Shengjie Tian, Shimin Chang, Yaogang Lu, Jianhua Zhu, Xuqiang Kong

**Affiliations:** 1 Department of Orthopaedics, Yangpu Hospital, Tongji University School of Medicine, No. 450, Teng Yue Road, Yangpu District, Shanghai, 200090 People’s Republic of China; 2 Department of Emergency Traumatic Surgery, Shanghai University of Medicine & Health Sciences Affiliated Zhoupu Hospital, Shanghai, 201318 China; 3 Department of Radiology, Shanghai University of Medicine & Health Sciences Affiliated Zhoupu Hospital, Shanghai, 201318 China

**Keywords:** Skeletal muscle, Intracompartmental pressure, Trauma, Tibial diaphyseal fracture, Magnetic resonance

## Abstract

**Background:**

Due to the special anatomy of the lower leg, tibial diaphyseal fracture causes increased intracompartmental pressure (ICP). Not only is this increased ICP the manifestation of skeletal muscle injury, but it induces further deterioration of the injury. The aim of this study was to assess the association between short-term ICP elevation and long-term skeletal muscle recovery after severe limb trauma.

**Methods:**

In this single-center ambispective cohort study, we retrospectively screened and recruited a cohort of tibial diaphyseal fracture patients with integrated ICP data during the early post-traumatic period, and performed a prospective observational study to evaluate their skeletal muscle recovery through long-term follow-up and MR imaging after the removal of the implants. We analyzed the association between ICP elevation and skeletal muscle recovery using statistical methods.

**Results:**

A total of 46 patients with healed fractures underwent intramedullary nail removal and MR imaging. The absolute values of the Pearson product-moment correlation coefficients between various ICP parameters and the cross-sectional area ratio (CSAR) ranged from 0.588 to 0.793, and the correlation coefficients between the ICP parameters and the average T2-weighted signal intensity ratio (T2SIR) varied from 0.566 to 0.775. Statistically significant associations were observed between the ICP parameters and the MR imaging parameters when simple linear regression analysis was performed. Among the ICP parameters, the accumulated Δ*P* (Δ*P* = diastolic blood pressure minus ICP) had the highest determination coefficient and explained 62.1% and 59.1% of the variance in CSAR and T2SIR, respectively.

**Conclusions:**

Short-term ICP elevation was associated with long-term skeletal muscle recovery following tibial diaphyseal fracture, especially for ICP data that integrated time factors.

**Level of evidence:**

Level 3.

## Introduction

Tibial diaphyseal fracture is a commonly encountered injury, accounting for approximately 2% of all fractures in orthopedic trauma [1–4]. Anatomically, the lower leg consists of four compartments. In a tibial diaphyseal fracture, bleeding, edema, and exudation are confined within those compartments, resulting in increased intracompartmental pressure (ICP) [5, 6]. In general, the more severe the trauma to the calf, the greater the energy transmitted to the soft tissues, leading to more extensive bleeding, edema, and exudation and hence higher pressure in the compartment [6, 7]. In addition to hindering blood circulation and causing the vicious cycle of ischemia–edema–ischemia aggravation, the increased ICP—the manifestation of skeletal muscle injury—also induces further deterioration of the injury [8]. The skeletal muscle is continuously damaged during the period of elevated ICP [9], such that an extended period of elevated ICP prevents full recovery from the injury [10]. Skeletal muscle that cannot be fully repaired ultimately degenerates and fibrosis repair occurs [11], which manifests as a decrease in skeletal muscle volume and a change in internal structure, affecting the patient’s physical performance in daily life and sporting activities [12]. Magnetic resonance (MR) imaging is recognized as one of the best diagnostic methods for skeletal muscle injury due its excellent resolution and its nonradiographic and noninvasive nature [13, 14]. MR imaging is a powerful tool for evaluating and quantifying skeletal muscle volume and internal structures [15, 16]. However, metal internal fixators such as intramedullary nails can interfere with MR imaging, which limits its application for evaluating the recovery of skeletal muscle in tibial diaphyseal fracture patients with intramedullary nails [17, 18].

Nevertheless, because of the influence of their cultural traditions and religious beliefs, our patients do not want implants in their bodies permanently; thus, in most cases, they volunteer to have the intramedullary nails removed when their fractures have completely healed. This meant that there was an opportunity for us to use MR imaging to assess the recovery of skeletal muscle following tibial diaphyseal fracture. Therefore, we retrospectively screened and recruited a cohort of tibial diaphyseal fracture patients with integrated ICP data in the early post-traumatic period. Furthermore, we conducted a prospective observational study to evaluate their skeletal muscle recovery through long-term follow-up and MR imaging after the removal of the implants. The purpose of the current study was to assess the association between short-term ICP elevation and long-term skeletal muscle recovery after severe limb trauma.

## Materials and methods

### Ethical approval

The present study was approved by the Ethics Committee of Shanghai University of Medicine & Health Sciences Affiliated Zhoupu Hospital (2017-C-003), and the patients were fully informed about the purpose and procedure of this study before they underwent ICP monitoring, intramedullary nail removal, and MR imaging. They consented to ICP monitoring, MR imaging, and voluntary removal of the intramedullary nail after fracture healing. All patients provided written informed consent before the procedure.

### Patient population

The current study was conducted as a single-center ambispective observational cohort study at a trauma center that provides medical care for approximately 1,000,000 community residents from March 2017 to July 2019. Patients with severe lower extremity trauma who were diagnosed with tibial diaphyseal fractures were retrospectively screened for possible recruitment. The inclusion criteria were as follows: (1) tibial diaphyseal fracture as defined by AO/OTA classification code 42; (2) fixation of the tibial diaphyseal fracture with intramedullary nails; (3) complete ICP data for the anterior compartment after the trauma, with the maximum ICP above 30 mmHg and the minimum Δ*P* (Δ*P* = diastolic blood pressure minus ICP) below 50 mmHg; (4) aged over 18 at the time of injury; and (5) the patient was willing to have the intramedullary nail removed after fracture healing. Based on the inclusion criteria, 64 patients were found to be eligible for the study.

**Fig. 1:**
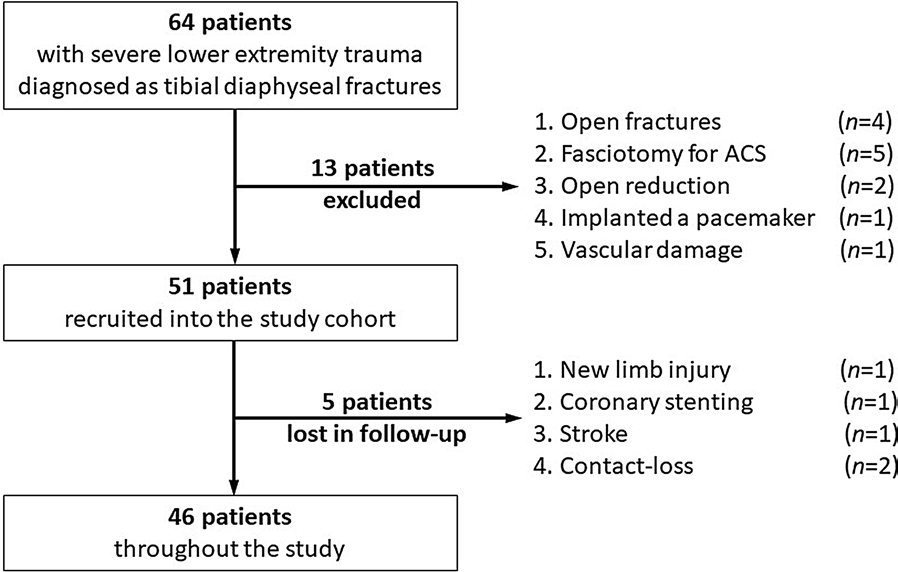
Flow diagram for patients throughout the study

The exclusion criteria were as follows: (1) open tibial diaphyseal fracture; (2) fasciotomy due to a diagnosis of acute compartment syndrome (ACS); (3) open reduction during surgery; (4) MR imaging contraindications (e.g., pacemaker); and (5) vascular and nerve damage to the affected limb. Finally, a total of 51 patients were recruited into the study cohort (Fig. 1).

### ICP data collection

**Fig. 2:**
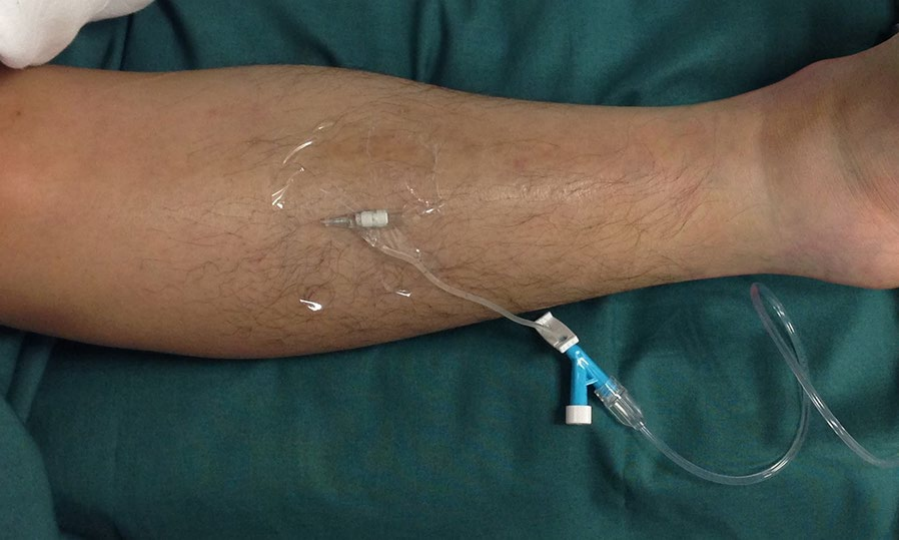
A 22-gauge intravenous catheter filled with normal saline was inserted into the anterior compartment of the affected limb and connected to an invasive arterial blood pressure monitor system (IABPMS) to measure and monitor the ICP continuously

In cases of severe calf trauma, such as tibial diaphyseal fractures, the anterior compartment is most frequently affected [8], so we measured and recorded the ICP in the anterior compartment immediately upon each patient’s arrival at the emergency room. A 22-gauge intravenous catheter filled with normal saline was inserted into the anterior compartment of the affected limb and connected to an invasive arterial blood pressure monitor system (IABPMS) to measure and monitor the ICP continuously for 48 h. The patient’s ICP and blood pressure were recorded by nurses each hour [19–22] (Fig. 2).

**Fig. 3:**
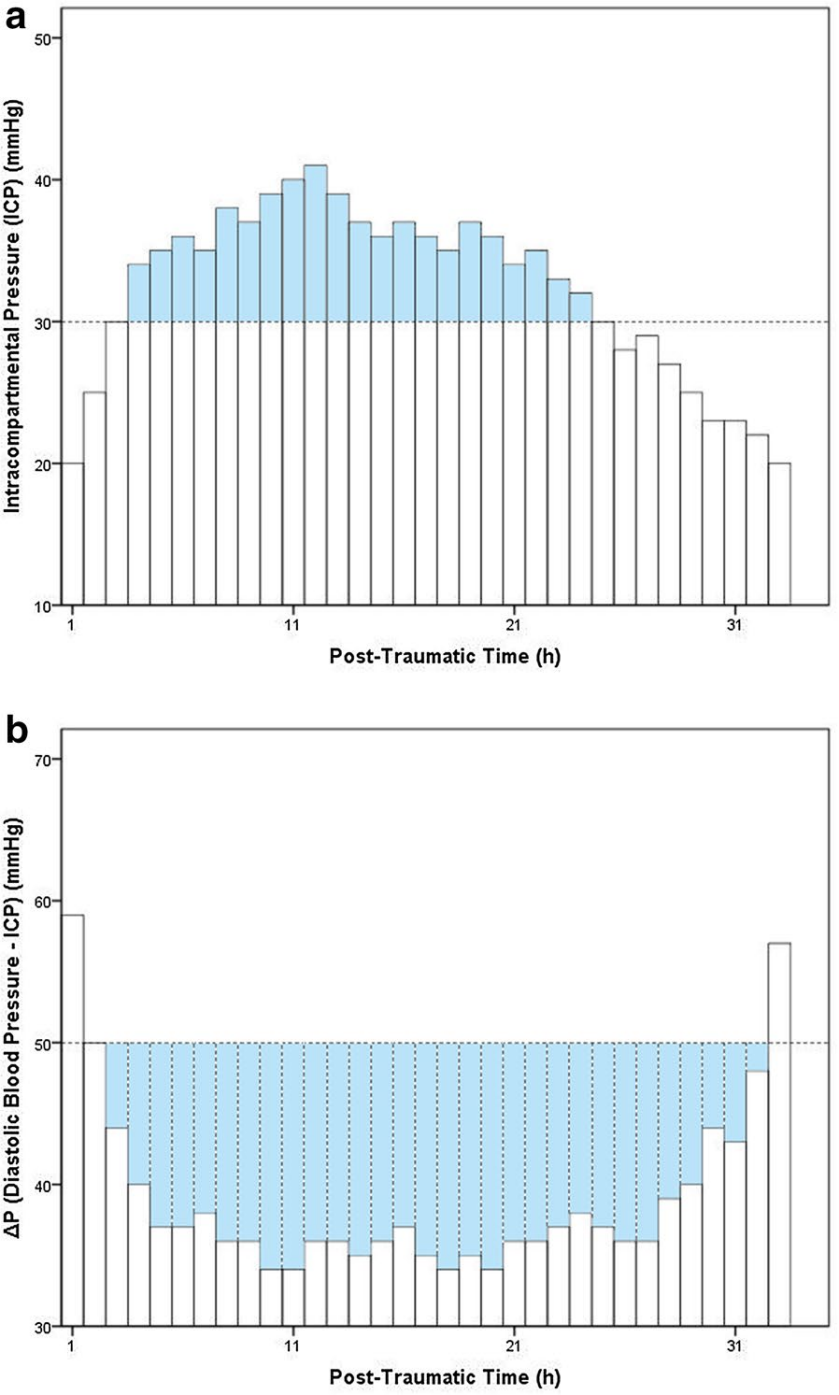
**a** Accumulated ICP: the sum of the hourly ICP values exceeding 30 mmHg. **b** Accumulated Δ*P*: the sum of the hourly Δ*P* values below 50 mmHg

Based on these records, we identified the maximum ICP and the minimum Δ*P*, which is a widely recognized indicator of skeletal muscle perfusion [21]. An ICP elevation of above 30 mmHg is considered clinically significant [5–7]. In order to determine the duration of ICP elevation based on continuous monitoring, the accumulated ICP was calculated as the sum of hourly ICP values exceeding 30 mmHg. However, simply adding Δ*P* values together is meaningless, because the smaller the Δ*P*, the more severe the obstruction of the blood circulation in the affected limb. According to a previous study, the diastolic blood pressure of patients increased drastically due to stress and pain after lower extremity injury, but gradually decreased with analgesia, immobilization, and hemorrhage, culminating in a mean diastolic blood pressure of about 80 mmHg 48 h after injury [19]. Since an ICP elevation > 30 mmHg was considered clinically significant and Δ*P* = diastolic blood pressure minus ICP, we determined a reference value of 50 mmHg. We then summed all of the hourly Δ*P* values below 50 mmHg (Fig. 3).

### Radiographic analysis

All patients were treated with closed reduction and intramedullary nail fixation for tibial diaphyseal fractures when the operating conditions permitted, and were subsequently followed up until bone union. Approximately 1 year after bone union was achieved, the patients requested the removal of the intramedullary nail; accordingly, we arranged a surgery after fully informing the patients of the risks involved and obtaining their informed consent. To avoid artifacts in the MR imaging from skeletal muscle edema and hemorrhage caused by the removal surgery, MR imaging was performed about 2 months thereafter using a high field strength (1.5 T) scanner (MAGNETOM Avanto Dot, Siemens Medical Solutions, USA). The patients laid supine in the body coil with their legs extended and relaxed so that images of the affected and normal limbs were obtained. Patients were imaged with axial and coronal turbo spin echo (TSE) T1-weighted sequences (TR 800 ms, TE 20–25 ms, SL 4–5 mm; in-plane resolution; matrix 512 × 512), TSE T2-weighted sequences (TR 2700 ms, TE 86 ms, SL 4–5 mm; in-plane resolution; matrix 512 × 512), and turbo inversion recovery magnitude (TIRM) T2-weighted sequences (TR 5510 ms, TE 32 ms, SL 4–5 mm; in-plane resolution; matrix 512 × 512). The acquired images were stored in Digital Imaging and Communications in Medicine format for further analyses.

**Fig. 4:**
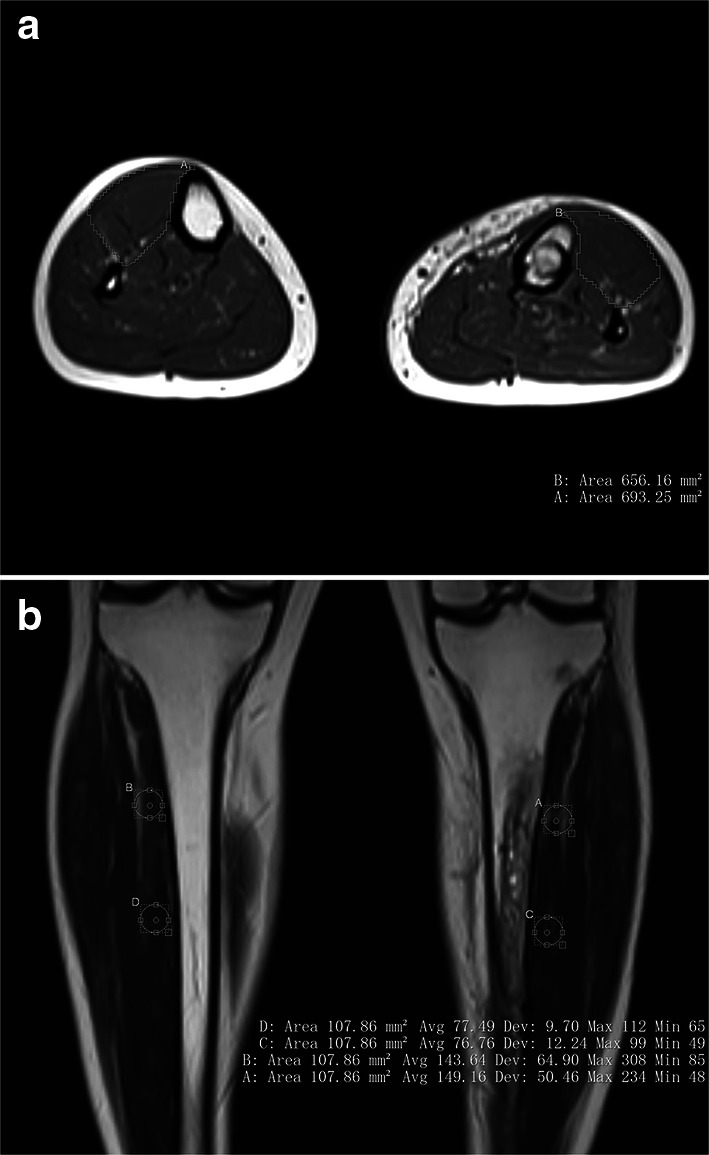
**a** The traced outlines of anterior compartments of the affected and normal limbs in the axial plane, which were used to measure anterior compartment cross-sectional areas (CSAs). The cross-sectional area ratio (CSAR) of the affected limb to the normal limb was calculated to evaluate the recovery of skeletal muscle volume. **b** T2-weighted image obtained in the coronal plane. Two regions of interest (ROIs), each with an area of 1 cm^2^, were chosen at identical locations in the affected and normal limbs . Vascular and fascial structures were kept outside the ROIs. The average T2-weighted signal intensity (T2SI) of the ROI was calculated for the affected limb and for the normal limb; the ratio of these T2SIs—the average T2-weighted signal intensity ratio (T2SIR)—was used to assess the recovery of skeletal muscle internal structures. Note the high-signal lesion in the medullary cavity of the affected tibia after the removal of the intramedullary nail

When the MR images were retrieved from the picture archiving and communication system (PACS), radiographic analysis of all cases was performed by a single radiologist who was blinded to the ICP data. Although it is considered to be the most accurate and reliable method for measuring skeletal muscle volume, MR imaging is costly and time-consuming when used for direct measurements [15, 23]. Therefore, a cross-sectional area (CSA) obtained from a single-slice image has been used in many studies as a quantitative indicator to evaluate skeletal muscle volume [12, 23, 24]. In the current study, we selected five slice cross-sectional images of bilateral calves and traced the outlines of the anterior compartments of the affected and normal limbs to measure the CSA of the anterior compartments. Using the CSA of the normal limb as a reference object, we calculated the ratio of the average anterior compartment CSA in the five slice images of the affected and normal limbs as the cross-sectional area ratio (CSAR) to assess the recovery of skeletal muscle volume (Fig. 4a). Furthermore, we selected five coronal plane slice images of the T2-weighted sequence, each of which contained the anterior compartments of the affected and normal limbs. We chose two regions of interest (ROIs) with an area of 1 cm^2^ for the anterior compartment of each limb in each image, thus selecting a total of 10 ROIs for each limb. As much as possible, the ROIs were chosen such that vascular and fascial structures were outside the ROIs and the ROIs were at identical locations for both limbs [25]. For each ROI, we calculated the average T2-weighted signal intensity (T2SI), after which the average value for 10 ROIs was accepted as the T2SI of the affected or normal limb (Fig. 4b). As in the case of CSAR, we compared the T2SI from the affected limb to that from the normal limb to obtain the average T2-weighted signal intensity ratio (T2SIR), which was used to assess the recovery of the internal structures of the skeletal muscle.

### Statistical analysis

The data were analyzed with SPSS® version 21.0 (SPSS Inc, Chicago, IL, USA). Continuous data are presented here as minimum–maximum (mean ± standard deviation). The Pearson product-moment correlation coefficient (PPCC) was used to investigate bivariate linear relations between the ICP data and the radiographic data. To evaluate the association of ICP elevation with the recovery of skeletal muscle, simple linear regression analyses of various ICP data and MR imaging data were performed. The significance level for all calculations was defined as *P* < 0.05.

## Results

In this ambispective cohort study, 51 patients met the criteria to be recruited, and a total of 46 patients underwent intramedullary nail removal and MR imaging after 11–30 (19.23 ± 5.01) months of follow-up. Five patients failed to meet the study criteria during follow-up because of a new limb injury, coronary stenting, stroke, and contact loss. All patients were treated with close reduction and intramedullary nail fixation when the operating conditions permitted, eventually achieving bone union. The fracture healing process lasted 15–46 (23.17 ± 5.82) weeks (Table 1). Two cases presented delayed union after intramedullary nail fixation; in those cases, the distal interlocking screws were removed and union was achieved after dynamization. The removal of the intramedullary nail was completely successful in all patients; there were no complications such intramedullary nails or screws remaining in the body, vascular or nerve injuries, hematoma, or infection.

**Table 1:** Demographics of the patients who were recruited and completed the study (*n* = 46)

Characteristic	Value
Age at injury (years)	22–72 (48.76 ± 15.46)
Female sex (#, %)	20 (43.5%)
Length of follow-up (months)	11–30 (19.23 ± 5.01)
Fracture union time (weeks)	15–46 (23.17 ± 5.82)
Time from initial surgery to nail removal (months)	9–29 (17.11 ± 4.93)
Time from nail removal to MR imaging (weeks)	6–11 (8.48 ± 1.53)
ICP data	
Maximum ICP (mmHg)	35–71 (48.33 ± 8.22)
Minimum Δ*P* (mmHg)	20–46 (36.33 ± 5.47)
Accumulated ICP (mmHg h)	21–298 (102.67 ± 60.11)
Accumulated Δ*P* (mmHg h)	13–203 (73.35 ± 40.99)
MR imaging data	
Cross-sectional area ratio (CSAR)	0.59–1.02 (0.85 ± 0.10)
T2-weighted signal intensity ratio (T2SIR)	0.98–1.63 (1.17 ± 0.15)

The 46 patients underwent MR imaging 6–11 (8.48 ± 1.53) weeks after the removal of the internal fixator. Measurements and calculations of the CSAR (0.59–1.02; 0.85 ± 0.10) and T2SIR (0.98–1.63; 1.17 ± 0.15) of the affected and normal limbs were obtained in all cases (Table 1). These showed that the CSAR presented a downward trend and the T2SIR an upward trend with increasing ICP.

**Fig. 5:**
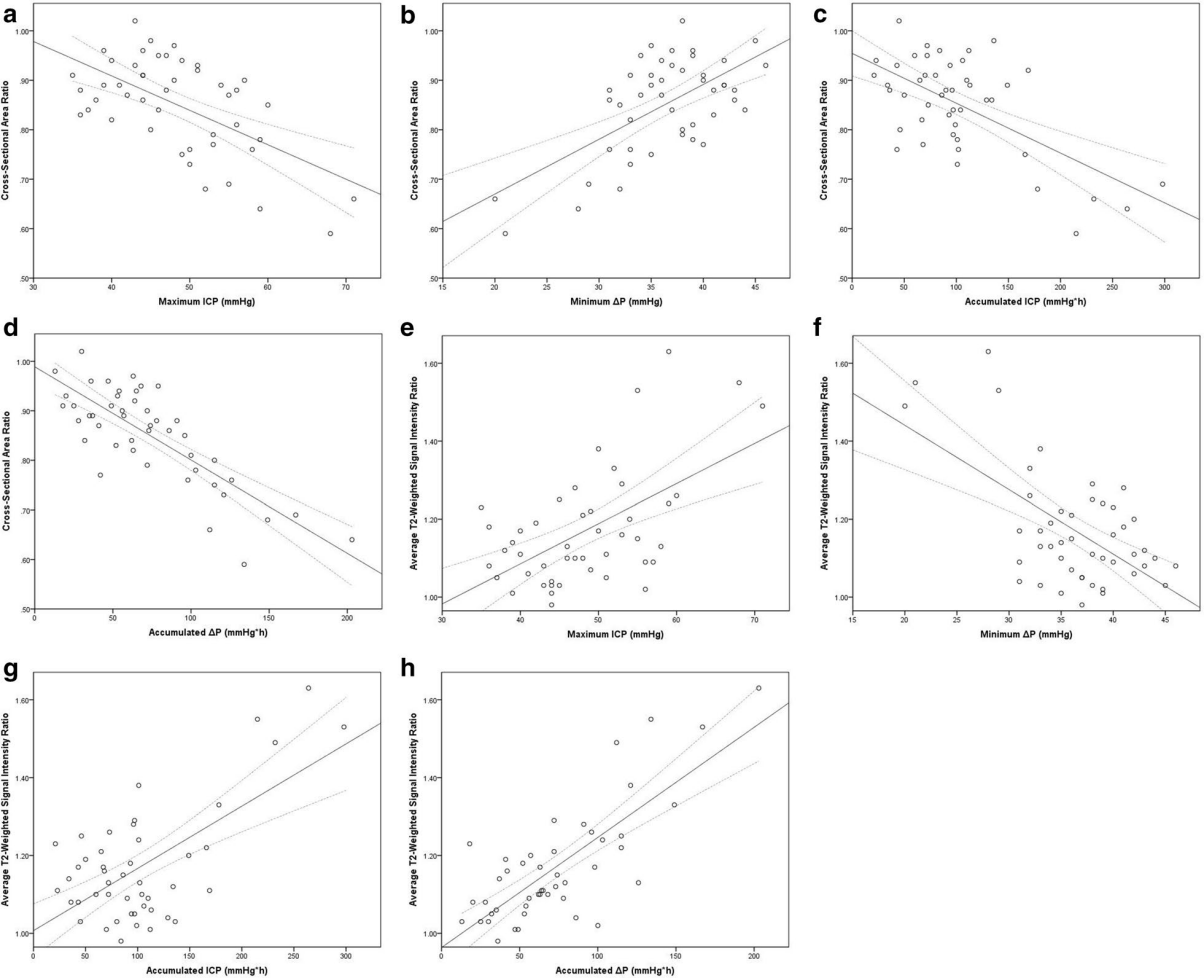
**a**–**d** Simple linear regression analyses of CSAR with the maximum ICP, minimum Δ*P*, accumulated ICP, and accumulated Δ*P*. **e**–**h** Simple linear regression analyses of T2SIR with the maximum ICP, minimum Δ*P*, accumulated ICP, and accumulated Δ*P*

The absolute values of the Pearson product-moment correlation coefficients between various ICP data and CSAR ranged between 0.588 and 0.793, while the correlation coefficients between various ICP data and T2SIR ranged between 0.566 and 0.775. Among all the ICP data, the accumulated Δ*P* showed the strongest correlations with CSAR and T2SIR (Table 2). Simple linear regression analysis showed that the maximum ICP, the minimum Δ*P*, the accumulated ICP, and the accumulated Δ*P* were statistically significantly associated with CSAR and T2SIR. The determination coefficients for the prediction of CSAR and T2SIR from the accumulated Δ*P* were the highest among all the ICP data (Table 3, Fig. 5).

**Table 2:** Pearson product-moment correlation coefficients (PPCCs) between the ICP data and MR imaging data

	Maximum ICP	Minimum Δ*P*	Accumulated ICP	Accumulated Δ*P*
Cross-sectional area ratio (CSAR)	− 0.588	0.623	− 0.622	− 0.793
T2-weighted signal intensity ratio (T2SIR)	0.566	− 0.604	0.642	0.775

**Table 3:** Results of simple linear regression analyses of the associations between the ICP data and the MR imaging data

	Cross-sectional area ratio (CSAR)				T2-weighted signal intensity ratio (T2SIR)			
	Regression coefficient	Constant	*R*^2^	SEE	Regression coefficient	Constant	*R*^2^	SEE
Maximum ICP	− 0.007	1.187	0.331	0.080	0.010	0.674	0.304	0.125
Minimum Δ*P*	0.011	0.448	0.375	0.076	− 0.017	1.771	0.350	0.121
Accumulated ICP	− 0.001	0.954	0.373	0.077	0.002	1.007	0.399	0.116
Accumulated Δ*P*	− 0.002	0.989	0.621	0.060	0.003	0.964	0.591	0.096

## Discussion

Movement is produced by the transmission of forces generated by skeletal muscles along a biomechanical chain from muscles to tendons to bones [14, 26–28]. The weakest link in this chain determines the level of function of the limb [14, 29]. As it is a driver of limb movement, skeletal muscle plays an important role in motor function [30]. In the current study, we conducted an ambispective observational cohort study based on MR imaging after the removal of implants to investigate the relationship between short-term ICP elevation and long-term skeletal muscle recovery after severe limb trauma. If a diagnosis of ACS is not established, it is believed that skeletal muscles will eventually repair themselves and regain good condition. Actually, this is a misunderstanding. Injured skeletal muscle goes through a series of coordinated and interrelated phases of healing, including degeneration, inflammation, regeneration, and fibrosis, which is an area that continues to present a challenge [31]. Clinically, a large number of fracture cases achieve bone union, but incomplete recovery of skeletal muscles leads to a partial loss of function and susceptibility to reinjury [31, 32]. In the presence of implants, however, it is difficult to make a correct evaluation of skeletal muscle recovery using the examination methods available. Thus, there are just a few clinical studies on skeletal muscle recovery, and the recovery was either derived from experimental studies or tested empirically in those studies [33, 34].

Several factors, both iatrogenic and noniatrogenic, can affect skeletal muscle recovery. The extent of skeletal muscle disturbance in open reduction varies with the surgical approach used, and an open fracture can have catastrophic consequences for skeletal muscle recovery in the event of infection. To focus on the impact of ICP and exclude interfering factors, we developed strict inclusion and exclusion criteria to limit our research subjects to those who had a closed tibial diaphyseal fracture and were treated with closed reduction and intramedullary nail fixation. In so doing, we not only excluded the iatrogenic impact of open reduction and internal fixation on skeletal muscle recovery but also avoided the noniatrogenic impact of infection caused by an open fracture. Furthermore, fasciotomy was reported to be a traumatic procedure associated with a set of complications including chronic pain, muscle weakness, and nerve injury [35]. Since fasciotomy for compartment decompression is an independent iatrogenic factor affecting skeletal muscle recovery, it was included as an exclusion criterion for our study.

Recently, however, some researchers have emphasized the importance of monitoring ICP, as indicated in a previously reported study where McQueen et al. [20] estimated the sensitivity and specificity of continuous ICP monitoring for the diagnosis of ACS, and proved—as expected—that it had high sensitivity and specificity for the diagnosis of ACS. Monitoring ICP can reduce both the delay before fasciotomy and the development of sequelae, as the appearance of clinical symptoms and signs lags behind the pressure changes. Long-term ischemia and hypoxia will cause serious consequences; however, skeletal muscle can tolerate short-term ischemia and hypoxia, which can, for instance, be caused by the application of a tourniquet to temporarily block limb blood flow for surgery. Prayson et al. [36] reported that 63% of participants had at least one compartment measurement that exceeded a single threshold of 45 mmHg, but none of them developed compartment syndrome or required a fasciotomy. Egol et al. [37] observed a similar dynamic, fluctuating process for ICP in their prospective cohort study. Through a retrospective analysis of the ICP parameters collected in our study, we found that ICP increased rapidly because of local bleeding and tissue edema after tibial diaphyseal fractures, and that ICP tended to stabilize or even recede with immobilization, the application of a cold compress, dehydration, and other medical approaches [32]. Similar to the results reported by Prayson and Egol, ICP reached an extremely high level (ICP > 45 mmHg) in some patients, but so long as the ICP quickly decreased, no typical symptoms of ACS were observed. As indicated by the MR imaging performed after long-term follow-up, there was no significant impact on the recovery of skeletal muscle. Thus, it is important for the evaluation of the impact of ICP on skeletal muscle to take time factors into account.

In addition to the maximum ICP and minimum Δ*P*, we organized and calculated the accumulated ICP and accumulated Δ*P* based on the duration of ICP elevation. The correlation analysis showed strong correlations of the two accumulative parameters with skeletal muscle recovery. Linear regression analysis reconfirmed the importance of the ICP elevation duration, as indicated by the evidence that the maximum ICP and minimum Δ*P* (which do not take time into account) were less strongly associated with skeletal muscle recovery, while the accumulated Δ*P* (which takes time into account) produced the highest determination coefficients for CSAR and T2SIR, explaining 62.1% and 59.1% of the variance in CSAR and T2SIR, respectively.

The primary limitation of our study was the small sample size, especially the small number of cases with sustained elevated ICP. Most of the cases with with sustained elevated ICP were diagnosed with ACS, meaning that they were excluded from the study cohort due to fasciotomy decompression. This could make the reliability of the estimated specificity of using ICP elevation to predict skeletal muscle recovery questionable in patients with high accumulated ICP and Δ*P*. MR imaging can assess skeletal muscle recovery, but the results may not always be accurate. Further investigations that focus on larger cohorts and use more accurate and comprehensive approaches to investigate skeletal muscle recovery are needed to explore the underlying mechanism for the impact of elevated ICP on skeletal muscle recovery as well as the functional implications for limbs.

## Conclusions

In summary, this ambispective cohort study demonstrated that short-term ICP elevation was associated with long-term skeletal muscle recovery, and that the accumulated Δ*P*, which accounts for both the ICP data and time, was the ICP parameter most closely associated with the recovery of skeletal muscle. Firm conclusions such as the accumulated Δ*P* threshold for skeletal muscle recovery could not be drawn from this study due to its size, but the study can provide ideas and thoughts for future research.

## Data Availability

All data generated or analyzed during this study are included in this published article’s supplementary information files.

## Abbreviations

ICP: Intracompartmental pressure
MR: Magnetic resonance
ACS: Acute compartment syndrome
IABPMS: Invasive arterial blood pressure monitoring system
TSE: Turbo spin echo
TIRM: Turbo inversion recovery magnitude
PACS: Picture archiving and communication system
CSA: Cross-sectional area
CSAR: Cross-sectional area ratio
T2SI: Average T2-weighted signal intensity
T2SIR: Average T2-weighted signal intensity ratio
PPCC: Pearson product-moment correlation coefficient
